# Mechanical Transmission of Protozoan and Helminthic Parasites by Synanthropic Flies in Sana′a, Yemen

**DOI:** 10.1155/japr/4095876

**Published:** 2026-05-25

**Authors:** Abbas Mohammed Ahmed Al-Azab, Yousef A. J. Fadladdin, Saeed M. N. Alasmari, Adel Mohammed Redhwan

**Affiliations:** ^1^ Biological Science Department, Faculty of Science, Sana′a University, Yemen; ^2^ Department of Biological Sciences, Faculty of Sciences, King Abdulaziz University, Jeddah, Saudi Arabia, kau.edu.sa; ^3^ Department of Biology, Faculty of Science and Arts, Najran University, Najran, Saudi Arabia, nu.edu.sa; ^4^ Department of Biology, Faculty of Science, Bursa Uludağ University, Bursa, Türkiye

**Keywords:** mechanical transmission, protozoan and helminthic parasites, synanthropic flies, Yemen

## Abstract

Nonbiting flies, particularly *Musca domestica* and *Sarcophaga haemorrhoidalis*, are widely recognized as potential mechanical vectors of intestinal parasites. This study investigated the role of these flies in transmitting parasitic organisms in Sana′a City, Yemen, where poor sanitation may facilitate such transmission. From June to August 2023, 300 flies were randomly collected from six locations in Sana′a City, including markets, garbage sites, fish‐selling areas, and butcher shops. Flies were trapped using sticky ribbons and sweep nets, then transferred to sterile tubes containing 3 mL of saline. Morphological identification was performed using standard keys. To isolate parasites, flies were washed in saline, centrifuged, and examined microscopically using iodine staining. The parasites were identified based on standard morphological characteristics. Of the 300 flies examined, 195 (65%) carried intestinal parasites. Infection was more prevalent in *M. domestica* (74%) than in *S. haemorrhoidalis* (45%). Nine parasitic species were identified on the external surfaces, comprising three protozoan cysts—*Entamoeba coli* (40%), *E. histolytica* (55%), *Giardia* spp. (53%)—and six helminths—*Ascaris lumbricoides* (28%), *Enterobius vermicularis* (20%), *Taenia* spp. (5%), *Hymenolepis nana* (35%), *Trichuris trichiura* (5%), and *Strongyloides stercoralis* larvae (3%). The highest infection rate (87%) was recorded in flies from Bab Al‐Yemen, whereas the lowest infection rate (20%) was recorded at the Sana′a University Campus. This study confirmed that nonbiting flies in Sana′a City can carry a range of intestinal parasites on their external surfaces, posing a public health risk. Improved hygiene and further studies on the carriage of internal parasites are warranted.

## 1. Introduction

Medically important insects/arthropods, including houseflies (*Musca domestica*) and cockroaches (*Periplaneta americana*), are significant mechanical vectors of intestinal parasites, facilitating the transmission of pathogens such as *Entamoeba histolytica*, *Giardia* spp., and *Taenia* spp [1]. Flies are crucial insects in medical and forensic research. Although they can be a nuisance and easily adapt to human habitats, they tend to spread pathogens, either mechanically or biologically, through their behavior [2–4]. Approximately 18,000 species of true flies belong to four families: Muscidae, Sarcophagidae, Calliphoridae, and Fanniidae, which are of particular importance to humans. Globally, houseflies, *M. domestica* (L), 1758, are the most common domestic flies, accounting for approximately 90% of all flies found in human homes [5, 6].

*M. domestica* is approximately 6–7 mm long, and has a 13–15‐mm wingspan, grayish color, and four dark longitudinal stripes along the back of the thorax, and it spreads foodborne illness [7, 8]. They are important mechanical vectors of several pathogens, particularly enteric bacteria and intestinal parasites. They have been implicated in the transmission of bacterial agents causing diseases such as cholera and typhoid fever, as well as protozoan and helminth parasites of medical and veterinary importance [9, 10]. Although many of these infections can also be acquired through contaminated food, water, hands, or person‐to‐person contact [11], houseflies contribute significantly to their spread by acquiring pathogens from sewage, garbage, and other contaminated substrates on their body surfaces and mouthparts, and subsequently transferring them to human and animal food [12]. Numerous pathogens can survive both internally and on the external surfaces of *M. domestica* for several hours and, in some cases, for up to 35 days after acquisition [9, 13]. Flesh flies are common and conspicuous, and most of these flies breed in carrion, dung, or decaying material [14]. The feeding and breeding habits of synanthropic flies make them important vectors of many pathogens of humans and domestic animals [15]. The filth fly, *S. haemorrhoidalis* (Diptera: Sarcophagidae), is a fly belonging to the family Sarcophagidae, found almost worldwide [16]. It is a large fly, measuring approximately 12 mm from head to tail, with an ash‐gray color and four longitudinal dark stripes on the thorax, along with dark and light square dots on the abdomen; it lacks the postscutellum, the sizable swelling beneath the scutellum on the thorax. It is characterized by large, dark red compound eyes [14, 17, 18]. Most flesh flies breed in carrion, dung, or decaying material [17]. These flies′ feeding and breeding habits make them significant vectors for numerous pathogens affecting humans and domestic animals [15]. Types of helminth eggs found on the body surface of flies that cause infection include *Ascaris lumbricoides*, *Trichuris trichiura*, *Enterobius vermicularis*, *Ancylostoma* spp., *Strongyloides* sp., and *Hymenolepis nana* [5, 6, 12]. Numerous studies have demonstrated that flies act as mechanical vectors for a wide range of infectious agents, including various developmental stages of helminth and protozoan parasites affecting humans. Their synanthropic behavior, frequent contact with organic waste, and close association with both humans and animals provide the necessary conditions to acquire and spread infectious agents. Lau et al. [9] reported the high prevalence and species diversity of synanthropic flies in Malaysia and how these flies frequently interact with humans and wildlife. This ecological overlap increases their chances to serve as epidemiological bridges between animal reservoirs and human hosts. In this regard, it was shown by Manandhar and Gokhale [10] that *M. domestica* acts as an important mechanical vector of gastrointestinal pathogens, as its feeding and breeding preferences favor the dispersal of enteric bacteria and intestinal parasites. Further, Graczyk et al. [15] confirmed the mechanical transmission of protozoan parasites like *Giardia lamblia*, *E. histolytica*, and *Cryptosporidium parvum* through direct contact with fecal matters and subsequent contamination of food and utensils. These observations, in concert, emphasize the fly‐mediated dispersal of pathogens as an epidemiologically important factor in both urban and rural environments. Beyond the domestic setting, flies that infest livestock and peri‐urban environments further spread intestinal parasites. Liu et al. [19] reported *A. lumbricoides* and *T. trichiura* on flies collected from Sudanese slaughterhouses, illustrating their potential in the transmission of zoonotic parasites. Similarly, Fetene and Worku [20] highlighted the importance of nonbiting cyclorrhaphan flies in unsanitary areas, whereas Getachew et al. [21], in turn, established a strong link between fly infestation levels and the prevalence of intestinal parasites among slum dwellers of Addis Ababa, Ethiopia. The results of these studies reiterate that fly density and environmental hygiene are critical determinants of parasitic disease transmission dynamics. At the mechanistic level, these vectors are morphologically adapted to enhance efficiency. Sukontason et al. [22] have also presented ultrastructural evidence for the presence of pulvilli, or adhesive pads, on the legs of different species in Calliphoridae, Muscidae, and Sarcophagidae, which allow attachment and efficient transmission of microbial propagules like spores, cysts, and eggs. These findings taken together explain the ecological behavior and morphological traits that result in making flies highly efficient mechanical transmitters of infectious agents across a range of ecosystems.

The traditional market, due to its provision of organic materials and waste, is a potential fly habitat. Houseflies′ sponge‐feeding and regurgitating behavior, combined with their access to food items, cutlery, kitchens, and humans, make them efficient disease transmitters. To the best of our knowledge, limited published data or no published data are available on the role of nonbiting flies as mechanical vectors for the transmission of parasites in Yemen. Therefore, this is the first study to evaluate the potential of *M. domestica* and *S. haemorrhoidalis* as mechanical vectors for the transmission of intestinal parasites in Sana′a city, Yemen.

## 2. Materials and Methods

### 2.1. Study Setting

**Figure 1:**
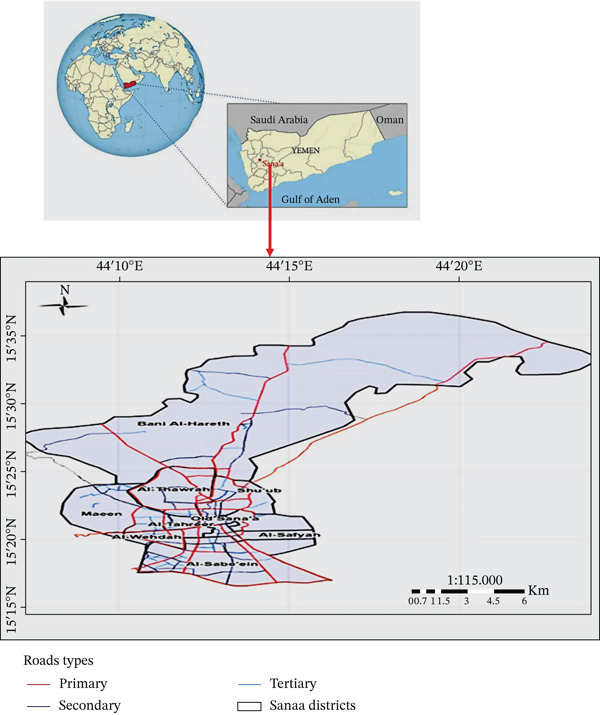
Map of Sana′a city showing study areas and fly sampling sites (Yemen Remote Sensing). The sources of these maps are as follows: Map obtained from *Journal of Tropical Medicine*. This material is subject to copyright, with terms and conditions applying. DOI: 10.1155/2022/5976640—Yemen Remote Sensing and GIS Center, Sana′a University.

The study was conducted in Amanat Al Assemah, Sana′a, the capital city of Yemen, located at approximately 2150 m above sea level. The geographic coordinates (15.356695°N, 44.200218°E) indicate the general location of Sana′a City rather than a specific sampling point. The climate is characterized by cold winters and moderate summers. Sampling was carried out at six representative locations distributed across Sana′a City: Al‐Tahreer (Bab Al‐Sabah), Al‐Safyah (Al‐Haraj), Hail Zone (20th Street), Madhbah (central market), Old Sana′a (Bab Al‐Yemen), and Sana′a University (campus) (Table 1 and Figure 1).

**Table 1:** Collection sites, GPS coordinates, and specific sampling points.

No.	Selected site	GPS coordinates	Specific sampling point
1	Al‐Tahreer (Bab Al‐Sabah)	15.354962°N	Fish‐selling areas, butcher shops, markets, and waste accumulation areas
		44.208999°E	
2	Assafi′yah (Al‐Haraj)	15.336192°N	Butcher shops, markets, and waste‐accumulation areas
		44.211024°E	
3	Hail zone (20th street)	15.355150°N	Butcher shops, markets, and waste accumulation areas
		44.187800°E	
4	Madhbah (central market)	15.398762°N	Markets, garbage sites and slaughterhouse‐associated environments
		44.180925°E	
5	Old Sana′a (Bab Al‐Yemen)	15.351247°N	Garbage sites, fish‐selling areas, and butcher shops
		44.215891°E	
6	Old Sana′a University (campus)	15.347825°N	Groceries and cafeterias
		44.189590°E	

These sites were selected purposively to represent areas with high human activity, varying sanitation conditions, and proximity to potential fly‐breeding and contamination sources, such as groceries, cafeterias, markets, waste accumulation areas, and slaughterhouse‐associated environments. This approach was adopted to capture a broad representation of urban settings within Sana′a City.

### 2.2. Collection of the Flies

Flies were collected weekly from 1 June to 30 August 2023 using trap‐night and a sweeping net at slaughterhouses, markets, garbage sites, fish‐selling places, grocery stores, cafeterias, and butcher shops in Sana′a city. Sampling was conducted at intervals rather than continuously. Sticky ribbons (Hunters Fly Catcher, made in China, plastic strips coated with a very sticky, nontoxic, nondrying adhesive, 3.5 cm wide and 55 cm long when fully unrolled) were used; three ribbons were placed at each selected site.

Three ribbons were hung at a height of 2 m above the ground and placed inside, or as close to, the entrances of the selected sites, along with sweeping the area with a net.

To increase the fly‐trapping surface, the ribbons were positioned vertically to avoid touching walls and were placed in shaded areas [10, 23].

The ribbons were kept exposed for 24 h (9:00 am–9:00 am) during each sampling period. Sweeping was carried out using an entomological sweep net, where the handle length of the net is 100 cm, made of wood, and the net bag, which is conical in shape, is 60 cm in length and 30 cm in diameter, and is fixed on the frame. The sweep net bag is cleaned and disinfected with 70% ethanol and dried to minimize cross‐contamination between sampling sites. The sweeping was conducted for 20 min at each sampling site during peak fly activity hours, morning (9:00–10:00) and late afternoon (16:00–17:00).

During the collection period, the minimum and maximum temperatures were recorded, with an average of 17°C and 32°C, respectively. Forceps were used to pick up individual flies from traps at each site and transfer them from the ribbons or nets into sterile collection tubes. The forceps were thoroughly cleaned with 70% alcohol and air‐dried completely between uses to prevent cross‐contamination. Next, 3 mL of normal saline was added to each tube, which was then labeled and transported to the Medical Entomology laboratory at the Department of Biological Sciences, Faculty of Sciences, University of Sana′a, for morphological identification.

### 2.3. Morphological Identification of Collected Flies

The collected flies were identified by an experienced medical entomologist, Dr. Abbas Al‐Azab, Associate Professor of Medical Entomology, Department of Biological Sciences, Faculty of Science, Sana′a University, Yemen, based on external morphological characteristics and standard taxonomic identification keys [9]. Specimens were examined under a stereomicroscope at 10×–40× magnification, focusing on diagnostic characters such as body colouration, thoracic and abdominal patterning, wing venation, and bristle arrangement. Species identifications were independently verified, and voucher specimens were preserved and deposited in the laboratory collection for future reference.

### 2.4. Identification of Parasites From the External Surfaces of Collected Flies

The collection tubes, containing flies and wash fluid (normal saline, 0.085 M), were shaken vigorously by hand to dislodge parasites from the external surfaces of the flies.

The washed fluids were transferred into a conical glass test tube and centrifuged at 2000 rpm for 5 min. The sediments were placed on clean glass slides, with or without Lugol′s iodine stain, and examined under a light microscope at 10× magnification to observe the parasites and 40× magnification for identification [12, 18, 24]. Slides were prepared in three replicates for each sample. The parasite stages were identified based on morphological details as described by [25, 26].

### 2.5. Data Analysis

The data collected from the field were first organized in Microsoft Excel. Subsequently, the data were analyzed using IBM SPSS Statistics for Windows (Version 29.0) [27] to assess the distribution of intestinal parasites detected in the collected fly specimens at the six sites. Before the analysis, the normality of the data distribution and homogeneity of variance were tested. Since the data did not show a normal distribution or homogeneity of variance, the analysis was performed using a nonparametric method. The results of the analysis, which included the presence of differences at various sites, were determined using the Kruskal–Wallis H test [28] to check if the differences were statistically significant. Descriptive results were also generated, showing the mean, standard deviation (SD), and median. A significance level of *p* ≤ 0.05 was used.

## 3. Results

**Figure 2:**
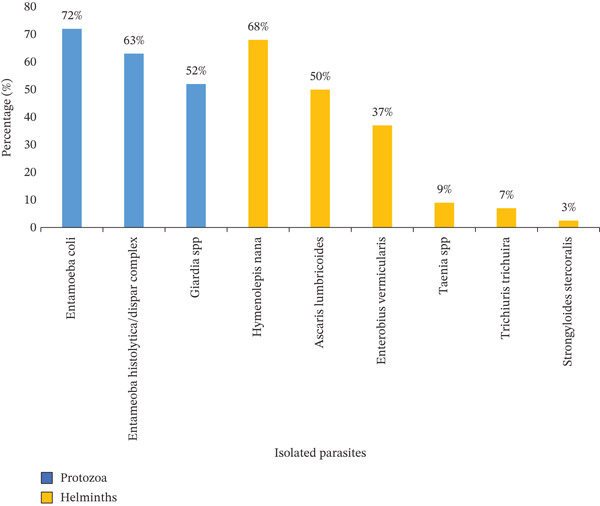
Prevalence of parasite species detected on the external surfaces of *M. domestica* (*n* = 211) in Sana′a city, Yemen, 2023. The percentages represent the proportion of the total number of examined flies (*n* = 211) carrying each specific parasite.

**Figure 3:**
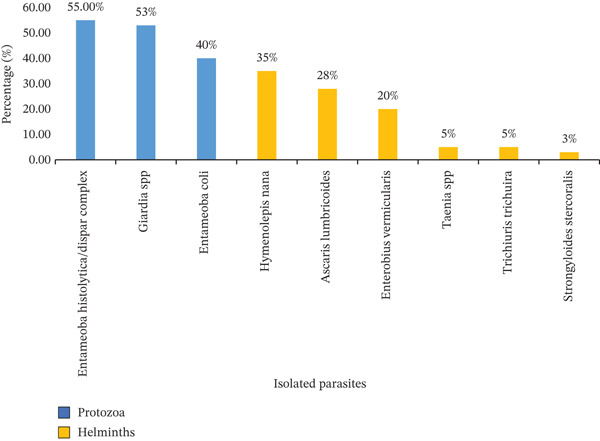
Prevalence of parasite species detected on the external surfaces of *S. haemorrhoidalis* (*n* = 89) in Sana′a city, Yemen, 2023. The percentages represent the proportion of the total number of examined flies (*n* = 89) carrying each specific parasite.

A total of 300 flies were collected and examined for intestinal parasites from six sampling locations in Sana′a City, Yemen, during the period from June to August 2023. The species identified in this study were *M. domestica* and *S. haemorrhoidalis*. Of the 300 samples, 195 (65%) carried at least one parasite stage on the external surface wash for parasites. Specifically, 155/211 (73%) *M. domestica* and 40/89 (45%) *S. haemorrhoidalis* carried ≥ 1 parasite stage (Tables 2, 3, and 4 and Figures 2 and 3).

**Table 2:** Percentage of intestinal parasites detected from *M. domestica* and *S. haemorrhoidalis* in Sana′a City, Yemen, 2023.

Selected areas	No. of collected flies	*M. domestica*		*S. haemorrhoidalis*		Total number of positive/contaminated flies
		No. of collected flies	Positive/contaminated *M. domestica* *n*/*N* (%)	No. of collected flies	Positive/contaminated *S. haemorrhoidalis* *n*/*N* (%)	
Al‐Tahreer (Bab Al‐Sabah)	55	40	33 (83%)	15	8 (53%)	41
Assafi′yah (Al‐Haraj)	65	40	34 (85%)	25	9 (36%)	43
Hail zone (20th street)	60	50	35 (70%)	10	6 (60%)	41
Madhbah (central market)	46	28	22 (79%)	18	7 (39%)	29
Old Sana′a (Bab Al‐Yemen)	34	23	20 (87%)	11	8 (73%)	28
Old Sana′a University (campus)	40	30	11 (37%)	10	2 (20%)	13
Total	300	211	155 (74%)	89	40 (44.94%)	195 (65%)

**Table 3:** Statistical parameters of parasite species detected from *Musca domestica* (*n* = 211) examined in Sana′a City, Yemen, 2023.

Parasite/selected areas	Statistical parameter	*Strongyloides stercoralis*	*Trichiuris trichuira*	*Tania spp*	*Enterobius vermicularis*	*Hymenolepis nana*	*Ascaris lumbricoides*	*Giardia sp*	*Entamoeba histolytica*/*dispar complex*	*Entamoeba coli*
Madhbah (central market)	Mean ± SD	0 ± 0	0 ± 1	1 ± 1	1 ± 1	3 ± 0	2 ± 1	2 ± 0	1 ± 0	3 ± 1
	Median	0	0	1	1	3	2	2	1	3
Al‐Tahreer (Bab Al‐Sabah)	Mean ± SD	0 ± 1	1 ± 1	0 ± 1	3 ± 1	7 ± 2	4 ± 1	4 ± 1	5 ± 1	5 ± 1
	Median	0	1	0	3	7	3	4	5	5
Old Sana′a University (campus)	Mean ± SD	0 ± 0	0 ± 0	0 ± 0	0 ± 0	1 ± 1	0 ± 1	0 ± 1	2 ± 1	1 ± 0
	Median	0	0	0	0	1	0	0	2	1
Hail zone (20th street)	Mean ± SD	0 ± 1	1 ± 1	1 ± 1	5 ± 3	9 ± 1	7 ± 3	7 ± 1	9 ± 1	11 ± 1
	**Median**	0	1	1	6	9	7	7	9	11
Old Sana′a (Bab Al‐Yemen)	Mean ± SD	0 ± 0	1 ± 1	1 ± 1	3 ± 2	4 ± 1	5 ± 1	6 ± 3	5 ± 1	7 ± 1
	Median	0	1	1	2	3	4	7	5	7
Assafi’yah (Al‐Haraj)	Mean ± SD	0 ± 1	1 ± 1	2 ± 1	8 ± 1	11 ± 1	9 ± 1	8 ± 1	11 ± 1	11 ± 2
	Median	0	1	2	8	11	9	8	11	11
	Kruskal–Wallis H	3.40	7.70	10.70	14.94	16.30	15.15	14.83	16.15	16.18
	*p* value	0.639	0.174	0.058 ∗	0.011 ∗	0.006 ∗	0.010 ∗	0.011 ∗∗	0.006 ∗	0.006 ∗

Abbreviations: H, Kruskal–Wallis test statistic; df, degrees of freedom (5).

∗Statistically significant at the *p* < 0.05 level.

**Table 4:** Statistical parameters of parasite species detected from *S. haemorrhoidalis* (*n* = 89) examined in Sana′a City, Yemen, 2023.

Parasite/selected areas	Statistical parameter	*Strongyloides stercoralis*	*Trichiuris trichuira*	*Tania spp*	*Enterobius vermicularis*	*Hymenolepis nana*	*Ascaris lumbricoides*	*Giardia sp*	*Entamoeba histolytica*/*dispar complex*	*Entamoeba coli*
Madhbah (central market)	Mean ± SD	0 ± 0	0 ± 0	0 ± 0	0 ± 1	0 ± 1	0 ± 1	1 ± 1	0 ± 1	1 ± 1
	Median	0	0	0	0	0	0	0	0	0
Al‐Tahreer (Bab Al‐Sabah)	Mean ± SD	0 ± 0	0 ± 0	0 ± 0	0 ± 1	1 ± 0	1 ± 1	1 ± 1	1 ± 1	1 ± 0
	Median	0	0	0	0	1	1	0	1	1
Old Sana′a University (campus)	Mean ± SD	0 ± 0	0 ± 0	0 ± 0	0 ± 0	0 ± 1	0 ± 1	0 ± 0	0 ± 0	0 ± 0
	Median	0	0	0	0	0	0	0	0	0
Hail zone (20th street)	Mean ± SD	0 ± 0	0 ± 0	0 ± 0	0 ± 1	1 ± 0	1 ± 0	2 ± 1	2 ± 1	2 ± 1
	Median	0	0	0	0	1	1	3	2	2
Old Sana′a (Bab Al‐Yemen)	Mean ± SD	0 ± 0	0 ± 0	1 ± 1	1 ± 1	2 ± 1	1 ± 1	3 ± 2	2 ± 1	3 ± 1
	Median	0	0	0	1	2	1	2	2	3
Assafi′yah (Al‐Haraj)	Mean ± SD	0 ± 1	1 ± 1	0 ± 0	0 ± 1	0 ± 1	0 ± 1	1 ± 1	0 ± 1	1 ± 1
	Median	0	0	0	0	0	0	1	0	1
	Kruskal–Wallis H	5.00	5.00	5.00	7.44	10.27	4.30	9.32	12.94	11.06
	*p* value	0.416	0.416	0.416	0.190	0.068	0.508	0.097	0.024∗	0.050∗

Abbreviations: H, Kruskal–Wallis test statistic; df, degrees of freedom (5).

∗Statistically significant at the *p* < 0.05 level.

Statistical analysis using the Kruskal–Wallis H test revealed significant differences in the distribution of parasites isolated from *M. domestica* across the six study sites. Specifically, the parasites *E. vermicularis*, *H. nana*, *A. lumbricoides*, *Giardia* sp., *E. histolytica*/*dispar* complex and *Entamoeba coli* (H = 14.83–16.30) were significantly different (*p* < 0.05), indicating nonuniform prevalence across geographic locations. *H. nana* and *E. coli* displayed the most pronounced variance (*p* = 0.006). In contrast, no significant differences were observed for *S. stercoralis* (*p* = 0.639) and *Trichiuris trichuira* (*p* = 0.174), suggesting a relatively homogeneous distribution of these species across all sites. *Tania* spp. showed borderline significance (*H* = 10.70, *p* = 0.058). These findings reveal heterogeneity in parasites occurrence across the selected areas, with six out of nine species (66.7%) exhibiting significant geographic variation. Meanwhile, the *E. histolytica*/*dispar* complex and *E. coli* (H = 12.94 and 11.06, respectively) were significantly different (*p* < 0.05), whereas no significant differences were observed for *Strongyloides stercoralis*, *T. trichuira*, *Tania* spp., *E. vermicularis*, *H. nana*, *A. lumbricoides*, and *Giardia s*p. (H = 4.30–10.27). The median values were utilized to represent the central tendency of the parasite counts, as the data exhibited a nonnormal distribution, providing a more robust measure against extreme outliers in certain study sites.

Parasites were primarily positive/contamination rate from houseflies, *M. domestica*, with 74% showing contamination compared with synanthropic flies, *S. haemorrhoidalis*, which had a 45% contamination rate. The study identified nine species of parasites on the external surfaces of flies. Three protozoan cyst taxa were detected. In *M. domestica*, the prevalence rates were as follows: *E. coli* (72%), *E. histolytica*/*dispar* complex (63%), and *Giardia* spp. (52%). In *S. haemorrhoidalis*, the corresponding rates were 40%, 55%, and 53%, respectively (Figures 2 and 3).

In addition, six helminth taxa (eggs and larvae) were identified. In *M. domestica*, the detected stages included *A. lumbricoides* (28%), *E. vermicularis* (20%), *Taenia* spp. (5%), *H. nana* (35%), *T. trichiura* (7%), and larvae of *S. stercoralis* (3%). Comparable helminth stages were also recovered from *S. haemorrhoidalis* (Figures 2 and 3).

*E. coli* was the most frequently detected taxon, being identified in 72% of the positive flies. In contrast, larvae of *S. stercoralis* showed the lowest prevalence (3%) among the positive samples.

Regarding sampling locations, overall (both species combined), the highest proportion of contaminated flies was recorded in Old Sana′a (Bab Al‐Yemen) (87%), followed by Al‐Safyah (Al‐Haraj) (85%). Conversely, the lowest infection rate was observed at Sana′a University (campus), where 20% of the examined flies carried at least one parasite stage (Table 2).

## 4. Discussion

Flies are considered nuisance insects and act as mechanical vectors in the transmission of various pathogens, such as parasites, to humans and animals, leading to human and zoonotic diseases [2, 29–33]. *M. domestica* is one of the most common fly species worldwide and is recognized as a vector for more than 100 pathogens [6, 19, 32, 33]. Our study′s results align with findings reported by Mohammad et al. [30] in Malakand, Pakistan; Khanet al. [34] in India; Al‐Hindi et al. [2] in Saudi Arabia; and Adenusi et al. [35] in Nigeria, where these studies identified *M. domestica* as a mechanical vector for transmitting several parasite species, such as *E. histolytica*, *Giardia* spp., *A. lumbricoides*, *T. trichiura*, *E. vermicularis*, *Ancylostoma caninum*, *S. stercoralis*, *and Taenia* spp. The results of this study showed that the two species of flies, *M. domestica* and *S. haemorrhoidalis*, have the potential to serve as mechanical vectors of parasites. These flies are attracted to human and animal feces for feeding and breeding, as they require proteins for egg development [22, 32].

The overall parasite carriage rate encountered in the present study, 65%, is considerably higher than in several studies carried out worldwide, while still lower than that reported in other studies. This prevalence is significantly higher than the global meta‐analytic estimate of about 43.3% presented by Ifedi et al. [4] and confirmed by Liu et al. [36], who estimated a similar rate for parasites of nonbiting flies globally. Additionally, it exceeds the estimated prevalence for the African subregion (~58.3%), indicating a comparatively higher level of environmental contamination in the current study area. Variability in parasite carriage among regions has been evident across multiple reports. For example, Balla et al. [6] investigated 1151 houseflies in Maiduguri, Nigeria, and detected gastrointestinal parasite ova and cysts on the external surfaces at relatively lower rates, 4.7%. Similarly, Jabal et al. [37] reported 2.6% prevalence of helminth eggs isolated from *M. domestica* in Makassar, Indonesia, whereas Hamoo and Alnuri [38] found a considerably higher prevalence (47%) among *M. domestica* in Mosul, Iraq. Addo et al. [39] also documented various intestinal parasites from synanthropic flies in Ghana, including protozoa such as *C. parvum* (95%) and *E. histolytica*/*dispar* (0. 83%), along with helminths including *A. lumbricoides* (3.34%) and *S. stercoralis* (0.83%), with an overall external contamination rate of 10.83%. Conversely, the current prevalence is lower than that reported in some studies from Iraq and Egypt, where markedly higher contamination levels have been observed. Al‐Aredhi [12] found that medically important parasites were isolated from both the external surfaces and digestive tracts of *M. domestica*, with an overall carriage rate of 81.3% (309/380) in Al‐Diwaniya province, Iraq, including protozoa (43.7%) and helminth eggs (37.6%). Similarly, Otu Bassey et al. [32] observed that the external body surface of *M. domestica* exhibited a significantly higher parasite frequency (76.7%) than their gut contents (16.7%) (*p* < 0.001). The highest contamination levels were reported by AbdAllah et al. [40] in Upper Egypt, who found infestation rates reaching up to 96.6% among certain fly pools, with *Cryptosporidium* detected in 64%–100% of samples and substantial contamination by *Entamoeba* and *Balantidium* species (22.6%–90.1% and 8.8.9%–100%, respectively), particularly in *M. domestica.*

Our findings are consistent with Hamoo and Alnuri. [38], who revealed that 11 types of parasites exist, with *E. histolytica* and *E. coli* comprising the majority. Our results are consistent with Ibrahim et al. [24], who reported that the identified intestinal parasites included cysts of *E. coli*, *E. histolytica*/*dispar*, *G. lamblia* (flagellate and cyst), *H. nana* eggs, and *Taenia species* eggs, with infectivity rates of 33.3%, 19%, 19%, 14.3%, 9.5%, and 4.8%, respectively, of the total house flies collected. Similar findings were reported by Ogunniyi et al. [33], who observed nine human intestinal parasites. *E. coli* (32.33%) was the most prevalent, followed by *A. lumbricoides* (15.79%). Garbage piles had the highest number of flies (324), whereas public latrines had the highest transmission rate (19.76%). Similar results were shown by Al‐Hindi et al. [2], who isolated various helminth eggs (e.g., *A. lumbricoides*, *T. trichiura*, *Taenia* spp., *E. vermicularis*, and *S. stercoralis*) and protozoan cysts/oocysts (*E. histolytica*, *E. coli*, *G. lamblia*, *Cryptosporidium* spp., and *Toxoplasma gondii*) from both external and internal structures of the flies. This supports the findings of El‐Sherbini and Gneidy [23], who discovered various intestinal protozoa and helminths in flies collected from food markets and hospitals in Egypt, highlighting their significant public health risks. The differences in the prevalence of parasites detected in the flies in this study compared with previous studies may result from waste accumulation around markets, sanitation constraints, and seasonal temperature. On the other hand, our results revealed that the percentage of protozoan parasites was higher than that of helminth larvae/eggs; these results are in agreement with Hamoo and Alnuri. [38] and with Otu‐Bassey et al. [32], who reported that the predominance of protozoan parasites in the external body surface of flies (13, 43.3%) over helminths (10, 33.3%), may suggest that most of these flies must have been from the insanitary locations to carry protozoa rather than helminths. The presence of *E. coli*, *E. histolytica*, and *G. lamblia* may be due to the ability of cysts and may contribute to fecal–oral transmission. These cysts can resist environmental conditions and survive for several weeks outside the host body, allowing them to reach flies [15, 41, 42]. Additionally, the polymeric coverage of the parasite generates hydrophobic, steric, and electrostatic attractive and/or repulsive forces [41].

In this study, the results demonstrated a higher rate of parasites detected on flies in the Old Sana′a (Bab Al‐Yemen), 87%, and Al‐Safyah (Al‐Haraj), 85% areas (Table 1), compared with other sites: Al‐Tahreer (Bab AlSabah), Hail zone (20th street), Madhbah (central market), and Old Sana′a University (campus). This variation may be attributed to the samples collected from traditional crowded markets located in unsanitary conditions, which provide organic materials and solid waste disposal nearby, creating a suitable environment for fly habitats and breeding, thus encouraging their survival and spread [38, 43]. In contrast, the lowest number of parasite stages was detected in flies collected from the Sana′a University campus. This finding may reflect comparatively better sanitation conditions and waste management practices in this area. Consequently, food sources were eliminated, and the environment was disinfected, preventing flies from picking up and spreading parasites.

Notably, the use of median values in the analysis of the data was a wise decision, especially in the presence of extreme outliers at some sites. These extreme outliers could be hotspots of parasite infection, and therefore should be targeted in future health interventions.

Finally, a discussion of the parasites isolated from *S. haemorrhoidales* and a comparison with those obtained from *M. domestica* could add another dimension to the study of parasite transmission in this region.

They possess certain biological features, such as a hairy and sticky exoskeleton that enables them to carry parasites [37, 44, 45], along with a feeding behavior that involves vomiting before licking food [46, 47]. These characteristics greatly enhance the transmission of parasites by flies. Commonly reported parasites of houseflies include the genera *Ascaris*, *Entamoeba*, *and Enterobius*, which are of significant medical and veterinary importance and cause enteric diseases such as amoebiasis, which is a deadly parasitic disease worldwide [48]. Additionally, houseflies are closely related to humans and domestic animals, and factors such as the availability of feces, the presence of parasites in fecal material, infected secretions, infectious agents carried by flies, and access to unprotected food and utensils all play crucial roles in disease transmission [10, 49].

## 5. Conclusion

This study emphasizes the epidemiological significance of *M. domestica* and *Sarcophaga haemorrhoidum*. The results of this study show that *M. domestica* flies are significantly more effective vectors of intestinal parasites than *S. haemorrhoidum* in Sana′a city, Yemen. However, differences in population density between the two fly species and environmental and hygienic conditions at the collection sites may partly explain the variations in prevalence. We recommend preventing these flies from contaminating human food, regularly removing dung, raising awareness about the importance of better hygiene, and enforcing strict sanitary measures. Implementing fly control programs should be a top priority to reduce fly populations and pathogen transmission in food markets and processing areas, helping to prevent potential outbreaks. Further research is needed to isolate and identify parasites from the digestive tracts and exoskeletons of various flies and to understand how these flies serve as vectors for contaminants from infected host sites.

## Funding

No funding was received for this manuscript

## Ethics Statement

The study protocol was reviewed and approved by the Animal Ethics Committee of the Biological Sciences Department, Faculty of Science, Sana′a University (Ethics Code: BAHSS105).

## Conflicts of Interest

The authors declare no conflicts of interest.

## Data Availability

The data used to support the findings of this study are included within the article and its supporting materials.
